# Natural Killer Repertoire Restoration in TB/HIV Co-Infected Individuals Experienced an Immune Reconstitution Syndrome (CAMELIA Trial, ANRS 12153)

**DOI:** 10.3390/pathogens12101241

**Published:** 2023-10-13

**Authors:** Polidy Pean, Yoann Madec, Eric Nerrienet, Laurence Borand, Didier Laureillard, Marcelo Fernandez, Olivier Marcy, Daniel Scott-Algara

**Affiliations:** 1 Immunology Unit, Institute Pasteur du Cambodge, Phnom Pen 12000, Cambodia; 2 Epidemiology of Emerging Diseases, Institut Pasteur, Université de Paris, 75000 Paris, France; yoann.madec@pasteur.fr; 3 Institut Pasteur, 75000 Paris, France; enerrienet@gmail.com; 4 Clinical Research Team, Epidemiology and Public Health Unit, Institut Pasteur du Cambodge, Phom Penh 12000, Cambodia; lborand@pasteur-kh.org; 5 Center for Tuberculosis Research, Division of Infectious Diseases, Johns Hopkins University School of Medicine, Baltimore, MD 20600, USA; 6 Infectious and Tropical Diseases Department, University Hospital, 30900 Nimes, France; didier.laureillard@chu-nimes.fr; 7 Médecins Sans Frontières, 1200 Geneva, Switzerland; msfch-lebanon-hom@geneva.msf.org; 8 Research Institute for Sustainable Development (IRD) EMR 271, National Institute for Health and Medical Research (INSERM) UMR 1219, University of Bordeaux, 33000 Bordeaux, France; olivier.marcy@u-bordeaux.fr; 9 Unité de Biologie Cellulaire et Lymphocytes, Institut Pasteur, 75000 Paris, France; daniel.scott-algara@pasteur.fr

**Keywords:** HIV, tuberculosis, NK cells, antiretroviral treatment, immune reconstitution, immune reconstitution inflammatory syndrome

## Abstract

IRIS is a common complication in HIV-infected patients treated for tuberculosis (TB) and cART. Our aim was to evaluate NK cell reconstitution in HIV-infected patients with TB-IRIS compared to those without IRIS. 147 HIV-infected patients with TB from the CAMELIA trial were enrolled. HIV+TB+ patients were followed for 32 weeks. The NK cell repertoire was assessed in whole blood at different time points. As CAMELIA has two arms (early and late cART initiation), we analysed them separately. At enrolment, individuals had low CD4 cell counts (27 cells/mm^3^) and high plasma viral loads (5.76 and 5.50 log/mL for IRIS and non-IRIS individuals, respectively). Thirty-seven people developed IRIS (in the early and late arms). In the early and late arms, we observed similar proportions of total NK and NK cell subsets in TB-IRIS and non-IRIS individuals during follow-up, except for the CD56dimCD16pos (both arms) and CD56dimCD16neg (late arm only) subsets, which were higher in TB-IRIS and non-IRIS individuals, respectively, after cART. Regarding the repertoire and markers of NK cells, significant differences (lower expression of NKp30, NKG2A (CD159a), NKG2D (CD314) were observed in TB-IRIS compared to non-IRIS individuals after the start of cART. In the late arm, some changes (increased expression of CD69, NKG2C, CD158i) were observed in TB-IRIS compared to non-IRIS individuals, but only before cART initiation (during TB treatment). KIR expression by NK cells (CD158a and CD158i) was similar in both groups. CD69 expression by NK cells decreased in all groups. Expression of the NCR repertoire (NKp30, NKp44, NKp46) has similar kinetics in TB-IRIS subjects compared to non-IRIS subjects regardless of the arm analysed. NK cell reconstitution appeared to be better in TB-IRIS subjects. Although NK cell reconstitution is impaired in HIV infection after cART, as previously reported, it does not appear to be affected by the development of IRIS in HIV and TB-infected individuals.

## 1. Introduction

Tuberculosis (TB) is a major cause of mortality and morbidity for individuals infected with the human immunodeficiency virus (HIV) worldwide [1]. The use of combined antiretroviral treatment (cART) for HIV-infected individuals results in a substantial reduction of HIV plasma viral load, improvement in CD4 T-cell counts, and restoration of the immune response and significantly reduces the risk of mortality and morbidity from opportunistic infections, including TB [2,3,4]. Although this immunological improvement correlates with beneficial outcomes and improved survival, a major complication, called immune reconstitution inflammatory syndrome (IRIS), can occur and is an obstacle for cART management [4,5]. Paradoxical TB-associated IRIS (TB-IRIS) is more common in high TB-prevalence countries, occurring in 4 to 54% of HIV/TB co-infected individuals who initiate cART during TB treatment [6,7]. TB-IRIS is believed to be associated with the impairment of inflammatory responses to *Mycobacterium tuberculosis (M. tb).* Rapidly expanding specific effector memory T-cells, along with the excessive release of pro-inflammatory cytokines and impairment of immune regulatory mechanisms to control the inflammatory reaction, have been suggested to be involved in the pathogenesis of TB-IRIS [8,9,10]. More evidence indicates the implication of innate immune effectors in the occurrence of TB-IRIS [8]. By using whole blood transcriptomic profiling, Lai et al. showed that the over-expression of innate immune mediators, including TLR, TREM-1 induced inflammasome signalling, caspase-1 and -5 and complement proteins related genes in monocytes/macrophages following two weeks post-cART were associated with TB-IRIS [11]. Also, the increased monocyte markers (soluble CD14 and CD163) and upregulated neutrophil activation transcript (S100 calcium-binding A9 and A8, the NLR family Pyrin domain containing 12, COX-1) were shown to be associated with the onset of TB-IRIS [12,13,14]. Studies by our team and a recent report have highlighted the importance of genetic factors in the physiopathology of IRIS [15,16], suggesting that IRIS may be a multifactorial syndrome.

The Cambodian Early versus Late Introduction of Antiretroviral (CAMELIA) clinical trial provided evidence that early introduction of cART (two weeks after TB treatment initiation) in HIV-infected patients significantly reduced mortality compared with later introduction (eight weeks after TB treatment initiation). However, this benefit was associated with an increased risk of TB-IRIS [4,17]. HIV-TB co-infected patients are less likely to have a better CD4 T cell count recovery on cART than those with HIV mono-infection. However, a significant increase in CD4 T cell count gain has been reported during the first year of cART treatment in individuals who experienced TB-IRIS compared with those who did not [17,18]. Therefore, the kinetics of early immune recovery may differ between IRIS and non-IRIS.

Natural killer (NK) cells are a key component of the innate immune system. They play a critical role in early host defence against viruses, tumour cells and intracellular bacteria, among other infections [19,20]. When activated by cytokines, pathogen-derived molecules or infected cells expressing ligands for NK cell receptors, NK cells become cytotoxic against target cells, secrete immunoregulatory cytokines and chemokines, and modulate other cellular immune responses. This includes the maturation of antigen-presenting cells to shape the magnitude and quality of the adaptive immune response [20]. These NK cell functions are regulated by a delicate balance of signals provided by activating and inhibitory receptors expressed on the surface of NK cells [20]. The NK cell response appears to play an important role in natural resistance to HIV infection and in the control of viral replication [21,22,23]. However, HIV infection can lead to NK cell dysfunction through the expansion of an abnormal CD16^pos^CD56^neg^ NK cell subset and modulation of the expression of several NK cell receptors, which can be at least partially restored by cART [24,25,26]. Similarly, TB infection can lead to suppression of the cellular immune response, including NK cells [27,28,29]. Partial recovery of NK cell cytotoxic activity and interferon-gamma (IFN-γ) has been reported after completion of TB treatment [27,28]. In addition, NK cells can kill *Mycobacterium tuberculosis*-infected monocytes and alveolar macrophages [30]. This killing is mediated by NK-activating receptors, NKp46 and NKG2D, which recognise vimentin and ULBP-1 expressed on the surface of infected cells [31]. Following activation by *M. tb*-infected monocytes, NK cells produce IFN-γ, which stimulates the production of IL-12, IL-15, and IL-18 by monocytes [31]. This, in turn, enhances IFN-γ production by CD8 T-cells [31]. IFN-γ is a principal cytokine that is involved in reactive nitrogen and oxygen-derived free radical synthesis and production by macrophages to kill intracellular bacteria [32]. Besides, memory-like NK cells induced by BCG-vaccine in humans and mice were reported to play a role in the immune protection against *M. tb* infection [33,34]. Moreover, the presence of HIV-specific-memory NK cells in rhesus macaques infected or vaccinated with simian immunodeficiency virus was also reported [35]. Altered NK cell activity has been reported in HIV/TB co-infection, and normal activity can be restored by in vitro addition of IL-12 and IL-15 [36].

In the CAMELIA clinical trial, we demonstrated that the capacity for NK cell degranulation, as defined by CD107a expression by exocytosis, was higher in TB-IRIS individuals than in those without TB-IRIS before cART initiation [37]. We also observed that the expression of the immunoglobulin-like receptor CD158a was higher in TB-IRIS individuals than in those without TB-IRIS [38]. This led us to hypothesise that individuals who develop TB-IRIS may have better NK cell reconstitution than those who do not develop TB-IRIS. Here, we compared the reconstitution of the NK cell repertoire in HIV/TB co-infected individuals who developed TB-IRIS with those who did not during early and late cART initiation, as well as in HIV-infected individuals without TB.

## 2. Individuals and Methods

### 2.1. Individuals

This study was linked to the CAMELIA clinical trial (ANRS 1295-CIPRA KH 001-DAIDS-ES ID 10425) [4]. From June 2006 to August 2008, 147 HIV-infected adults with TB (HIV+TB+) who were enrolled in the CAMELIA trial gave their specific signed consent to enter this study. HIV + TB+ individuals provided blood samples for NK immune-phenotyping assays at week 2 (i.e., cART initiation in the Early arm) and at week 8 (i.e., cART initiation in the Late arm), 14, and 34 after TB treatment initiation. Paradoxical TB-IRIS was defined as a documented clinical worsening of TB signs or symptoms during anti-TB treatment after cART initiation, which was not explained by any other diseases or an adverse effect of drug therapy; lymph node enlargement with inflammatory signs was considered IRIS. Each IRIS case was independently validated by experienced physicians of CAMELIA’s clinical coordinator team [4,17]. CD4 T-cell counts and plasma HIV RNA viral load, determined in a central laboratory of the Institute Pasteur du Cambodge, were available at enrolment and at weeks 26 and 50 after TB treatment initiation. The Cambodian National Ethics Committee for Human Research and the clinical ethics committee of the Pasteur Institute in Paris approved this study.

### 2.2. Phenotypic Studies of NK Cells

All phenotypic studies were performed on fresh whole blood using a four-colour BD FACScalibur II cytometer (BD, Paris, France). Flow cytometry data were acquired and collected by BD CellQuest Pro^TM^ and were analysed using FlowJo version 5.0. Lymphocytes were gated in the FSC/SSC dot plot, and NK cells were identified as CD3 negative and CD56 positive, and/or CD16 positive lymphocytes. NK-cell subsets were identified by co-expression of CD56 and CD16 on NK cells: CD56^bright^CD16^neg^ (CD56^++^CD16^−^) NK cell, CD56^dim^CD16^neg^ (CD56^+^CD16^−^), CD56^dim^CD16^pos^ (CD56^+^CD16^+^), and CD56^neg^CD16^pos^ (CD56^−^CD16^+^).

NK-cell natural cytotoxic receptors: NKp30 (CD337), NKp44 (CD336), NKp46 (CD335), and NK-cell Ig-like receptors (KIR): KIR2DL1/KIR2DS1(CD158a/h), and KIR2DS4 (CD158i), CD160, NKR-P1A (CD161), NKG2A (CD159a), NKG2C(CD159c), NKG2D (CD314), DNAM-1 (CD226), IL-2Rβ (CD122), 2B4 (CD244), and the activation marker CD69 were detected using monoclonal antibodies from Beckman Coulter (Paris, France), RD Systems (Paris, France) and BD Biosciences (Paris, France). The list and the combination of fluorochrome-conjugated monoclonal antibodies used for immune-phenotyping are shown in Supplementary Table S1. The gating strategy of NK cells is shown in the Supplementary Figure S1.

### 2.3. Statistical Analysis

CD4 T cell responses to cART at 26 and 50 weeks of follow-up were compared between IRIS and non-IRIS HIV+TB+ individuals using the Mann-Whitney non-parametric test. At the start of cART, total NK cells, NK cell subsets and NK cell repertoire were compared between TB-IRIS and non-IRIS using the Mann-Whitney U test. Measurements were taken at week 0, week 6, week 12 and week 32 after cART initiation for the HIV+TB+ individuals who initiated cART after two weeks of anti-tuberculosis treatment (i.e., Early cART initiation arm) and at week 6, week 0, week 6 and week 12 after cART initiation for HIV+TB+ individuals of the Late cART initiation arm. The evolution of NK cells and NK cell repertoire expression under cART of the matched timepoint was compared between TB-IRIS and non-IRIS patients using the Mann-Whitney U test. Statistical analysis was performed using GraphPad Prism version 6.0, GraphPad Software, San Diego, CA, USA, Statistical significance was defined as a *p*-value < 0.05.

## 3. Results

### 3.1. Clinical and Laboratory Features of Enrolled HIV-Infected Individuals

Of the 147 HIV + TB+ individuals from the CAMELIA trial originally enrolled in this study, 19 were excluded from the final analysis for the following reasons: ten individuals had no follow-up data on cART; five had suspected TB-IRIS that was not confirmed after the case validation; two had paradoxical TB reactions prior to cART initiation, and two started cART before the scheduled date.

At cART initiation, the demographic and clinical characteristics of all HIV+TB+ individuals (IRIS and non-IRIS) were similar. However, HIV + TB+ individuals had significantly lower CD4 T cell counts at cART initiation than HIV + TB− individuals, as described in our previous publication [37]. With cART, virological control, as measured by the proportion of individuals with undetectable HIV viral load, did not differ between TB-IRIS and non-IRIS individuals, as observed for the entire CAMELIA cohort [4,17].

**Figure 1:**
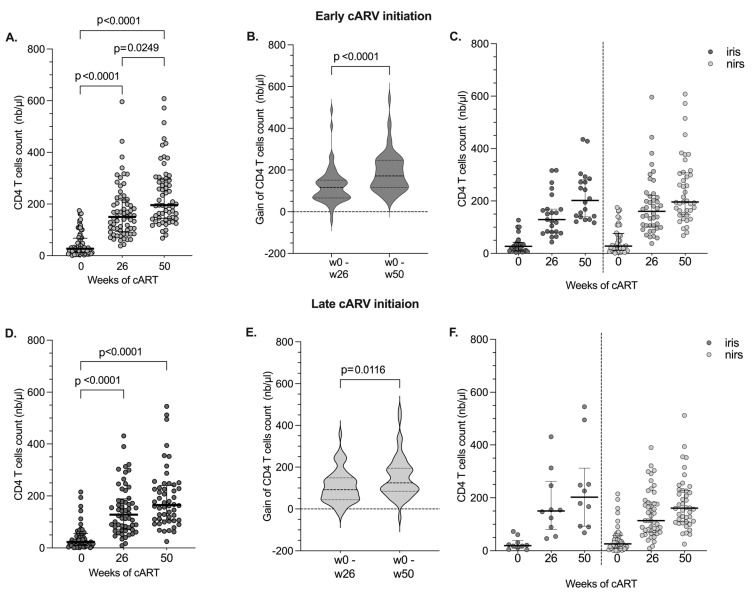
Reconstitution of CD4 T cell counts in HIV/ TB co-infected individuals according to enrolment in early and late cART initiation. Absolute CD4 T cell counts in HIV/TB co-infected individuals according to early (**A**) or late (**D**) initiation of cART at different time points. Increase in CD4 T cell count in early (**B**) and late (**E**) cART initiation arms from week 0 to week 26 and week 50. Absolute CD4 T cell counts in TB-IRIS and non-IRIS patients in the Early (**C**) or Late (**F**) cART initiation groups measured at enrolment, week 26 and week 50 of cART. The graph shows median values (25–75% IQR). Statistically significant differences (*p*-value < 0.05) are indicated.

For the next analysis, and because cART treatment was started at different times, TB-IRIS and non-IRIS individuals were separated according to early or late cART initiation. CD4 T cell reconstitution is shown in Figure 1. Significant increases in CD4 T cell counts were observed during follow-up in both arms (Figure 1A,D), as well as CD4 T cell gains at W26 or W50 (Figure 1B,E). In addition, similar CD4 T cell reconstitution was observed in both arms (Figure 1). When we analysed the CD4 T increase during follow-up according to IRIS or non-IRIS conditions in both arms, non-significant differences were observed (Figure 1C,F).

### 3.2. Analysis of the Immune Reconstitution of Peripheral NK Cells and Their Subsets in IRIS and Non-IRIS Individuals Enrolled in the Early and Late Arms of the Trial

**Figure 2:**
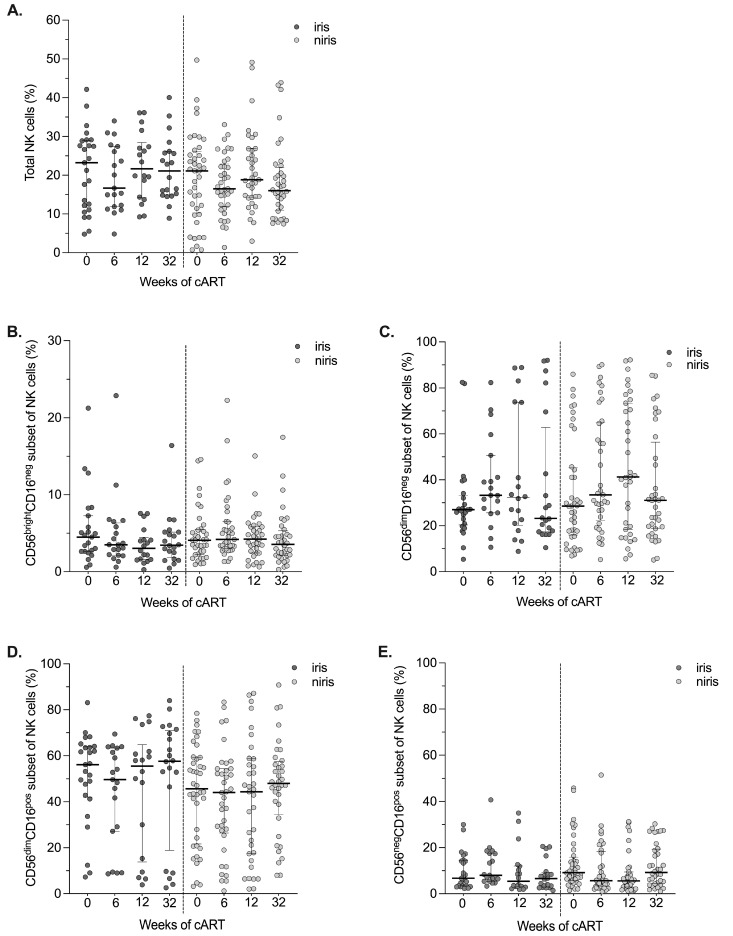
Changes in the total number of NK cell counts and their subsets in the early cART introduction group (Early arm) by IRIS occurrence. Proportion of total circulating NK cells (**A**) and their subsets: CD56^bright^CD16^neg^ (**B**), CD56^dim^CD16^neg^ (**C**), CD56^dim^CD16^pos^ (**D**), CD56^neg^CD16^pos^ (**E**) NK cells at baseline (week 0) and after 6, 12, 32 weeks of cART in TB-IRIS and non-IRIS HIV/TB co-infected individuals. Results are presented as median values (25–75% IQR). The *p*-value < 0.05 is statistically different and significant.

**Figure 3:**
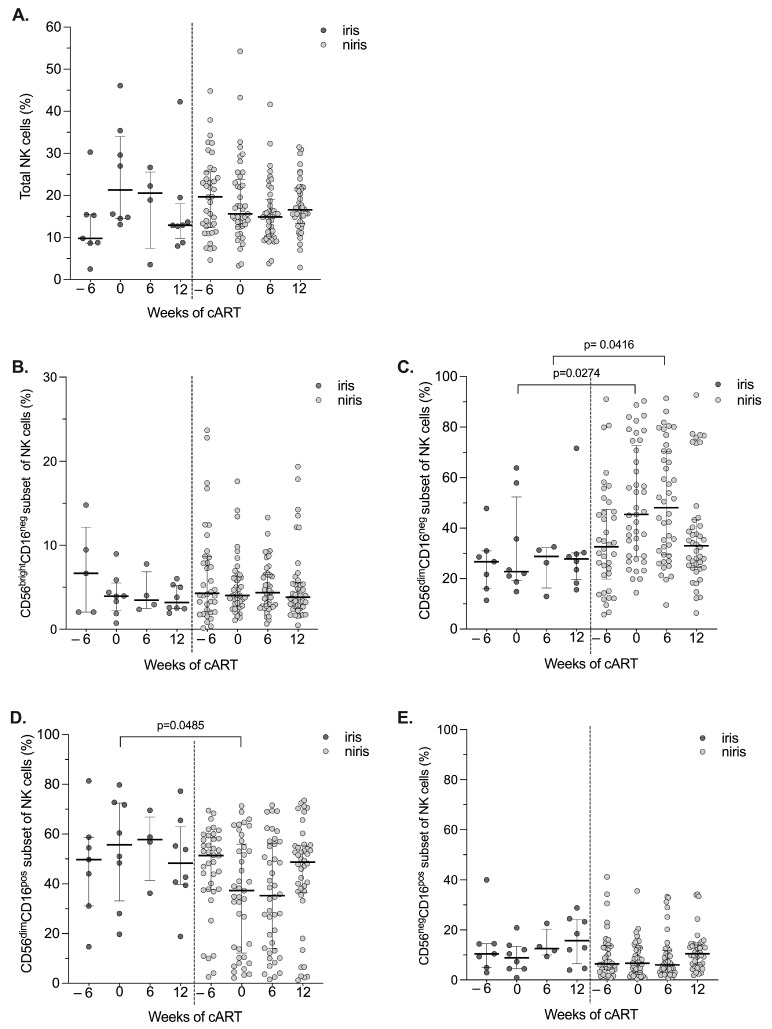
Changes in the total number of NK cells and their subsets in the late cART initiation group (Late arm group) according to the onset of IRIS. Proportion of peripheral blood NK cells (**A**) and their subsets: CD56^bright^CD16^neg^ (**B**), CD56^dim^CD16^neg^ (**C**), CD56^dim^CD16^pos^ (**D**), CD56^neg^CD16^pos^ (**E**) NK cells at baseline (week 6) and at 0, 6, 12 weeks of cART in TB-IRIS and non-IRIS HIV/TB co-infected individuals. Results are presented as median values (25–75% IQR). The *p*-value < 0.05 is statistically different and significant.

As impairment of NK cell reconstitution (total population and NK cell subsets) and persistence of NK dysfunction during chronic infection is described during cART, we would like to know whether the NK cell repertoire is affected by IRIS after initiation of cART. We first assessed the proportion of total peripheral NK cells and subsets, defined by CD16 and CD56 expression, on cART in TB-IRIS and non-IRIS individuals (Figure 2 and Figure 3). As mentioned above, we analysed people enrolled in the early and late arms separately because the introduction of ARVs is not the same in terms of the start of anti-tuberculosis treatment.

Similar percentages of total NK cells and NK cell subsets and kinetics during follow-up were observed in IRIS and non-IRIS individuals enrolled in the early arm (Figure 2 and Figure S4). In addition, as shown in Figure 2, the percentages of total NK cells and subsets are very stable during follow-up. In contrast, when cART was started eight weeks after TB treatment in the late arm (Figure 3 and Figure S5), several differences were observed between IRIS and non-IRIS individuals in both percentages and kinetics of NK cells, in particular NK cell subsets (Figure 3C,D and Figure S5C,D). Changes observed before the start of cART (between week 6 and week 0, late arm) could be related to anti-TB treatment. After starting anti-TB drugs, the CD56^dim^ CD16^pos^ NK population increases in IRIS individuals along with total NK cells and decreases in non-IRIS individuals (Figure S5A,D), whereas the CD56^dim^CD16^neg^ NK subset increases in non-IRIS and is stable in TB-IRIS (Figure S5C). These differences persist after the start of antiretroviral therapy. However, they are no longer observed at the end of follow-up (W12 after initiation of cART).

### 3.3. Analysis of Activation Marker and KIR Repertoire in IRIS and Non-IRIS Individuals Enrolled in Early and Late Arms

The CD69 marker on NK cells was used to track activation. Monitoring of this marker is important because immune activation decreases after cART initiation. The results are shown in Supplementary Figure S2 (early arm) and S3 (late arm). In the early arm and as expected, CD69 marker expression decreased with similar kinetics during follow-up in both IRIS and non-IRIS individuals (Supplementary Figure S2A,B).

Analysis of NK cell activation in the late arm group showed no differences in CD69 levels between W-6 and W0 (TB treatment prior to cART), irrespective of IRIS status (Supplementary Figure S3A,B). A decrease in CD69 expression on NK cells after cART initiation is observed in a similar manner in TB-IRIS and non-IRIS individuals. However, the decrease is higher in non-IRIS than in IRIS at W6 (Supplementary Figure S3A).

Regarding the KIR repertoire, the evolution of CD158a and CD158i expression on NK cells of the early and late arms are shown in Supplementary Figures S2 and S3. The evolution and kinetics of both receptors are similar and do not differ in the early or late arms in TB-IRIS and non-IRIS individuals.

### 3.4. Evolution of Lectin Receptors in IRIS and Non-IRIS Individuals

**Figure 4:**
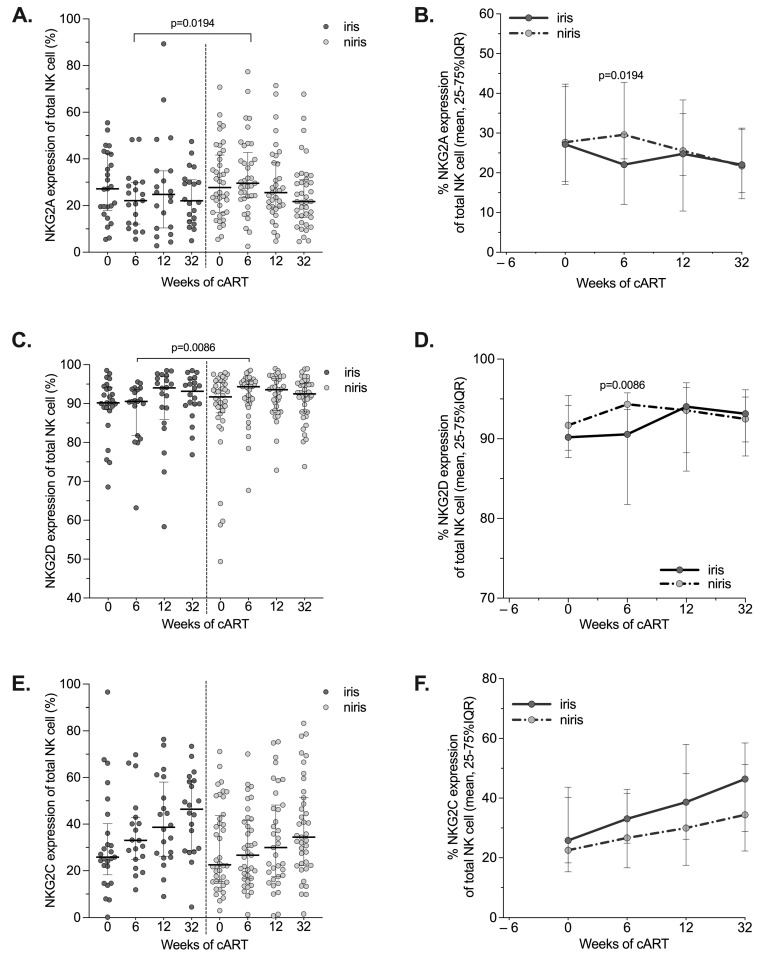
Changes in the expression of NKG2A/CD159a, NKG2C and NKGD2A on NK in the early cART introduction group (Early arm) by IRIS occurrence. The proportion and longitudinal changes of peripheral blood NK cells expressing NKG2A (**A**,**B**), NKG2D (**C**,**D**) and NKG2C (**E**,**F**) at baseline (week 0), week 6, 12 and week 32 of cART in TB-IRIS and non-IRIS HIV/TB co-infected individuals. Results are presented as median values (25–75% IQR). The *p*-value < 0.05 is statistically different and significant.

**Figure 5:**
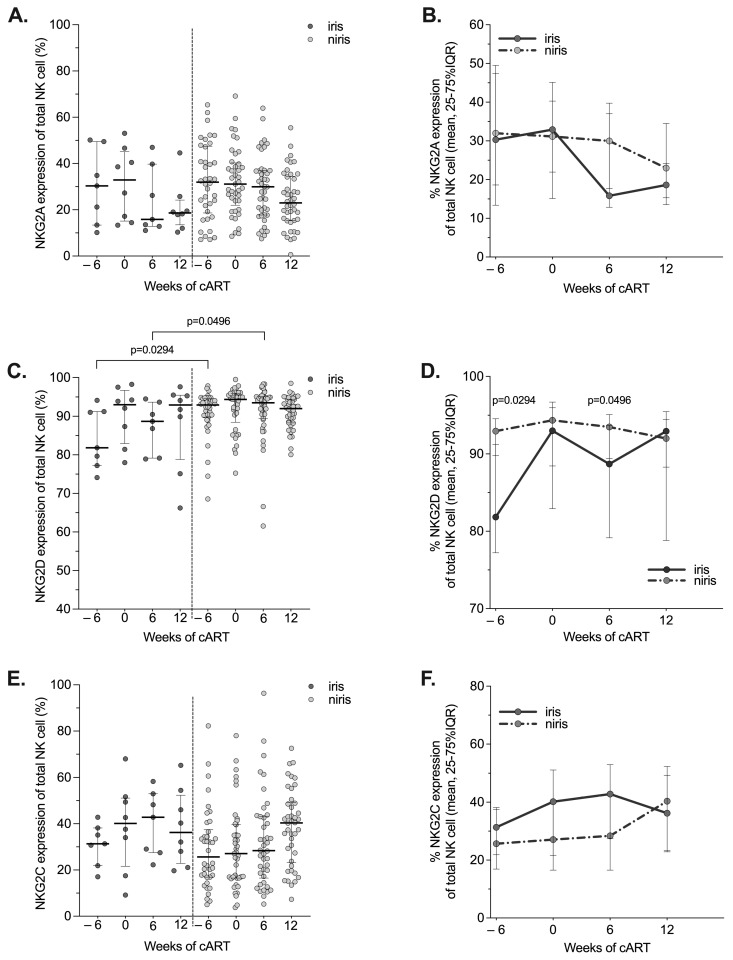
Changes in the expression of NKG2A/CD159a, NKG2C and NKGD2A on NK cells in the Late initiation cART group according to the onset of IRIS. Proportions and longitudinal changes of peripheral blood NK cells expressing NKG2A (**A**,**B**), NKG2D (**C**,**D**) and NKG2C (**E**,**F**) at baseline (6 weeks before cART initiation), week 0, week 6 and week 12 of cART in TB-IRIS and non-IRIS HIV/TB co-infected patients. Results are presented as median values (25–75% IQR). Statistically significant differences (*p*-value < 0.05) are indicated.

The results for the NK cell receptors of the C-type lectin-like family (NKG2A, NKG2D and NKG2C) are shown in Figure 4 and Figure 5. In the early arm, the lowest levels of NKG2A (CD159a) and NKG2D expression were observed in IRIS subjects compared to non-IRIS subjects at week 6 of cART (Figure 4A,C). Furthermore, the levels of both receptors were comparable in IRIS and non-IRIS and remained stable during follow-up (Figure 4B,C). Regarding the expression of NKG2C, the expression increased with similar kinetics regardless of the IRIS condition during the follow-up (Figure 4E,F).

The expression levels of CD85j, CD160, CD226 and CD244 increased, and CD161 decreased in NK cells during the observation period, but there were no differences between the groups and arms studied.

### 3.5. Expression of Natural Cytotoxic Receptors (NCRs) in IRIS and Non-IRIS during the Follow-Up

**Figure 6:**
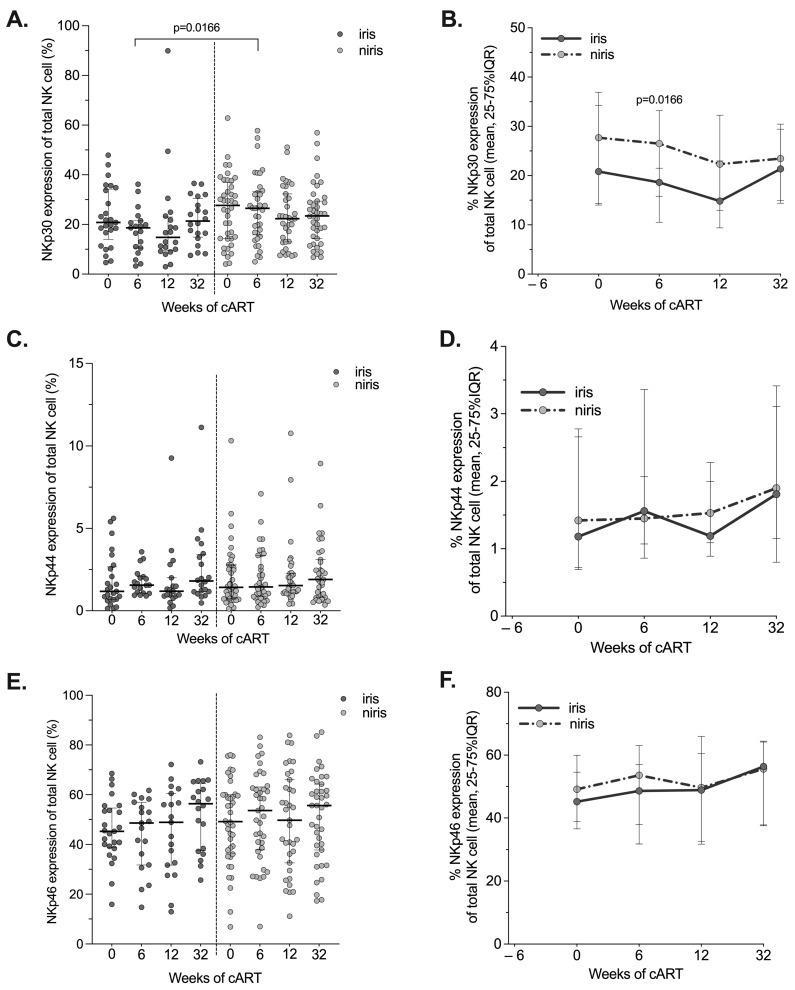
Changes in the expression of NCRs (NKp30, NKp44, NKp46) on NK cells in the early cART introduction group according to the onset of IRIS. Proportion and longitudinal changes of peripheral blood NK cells expressing NCRs: NKp30 (**A**,**B**), NKp44 (**C**,**D**) and NKp46 (**E**,**F**) at the time of the enrolment (week 0), week 6, 12 and week 32 of cART in TB-IRIS and non-IRIS HIV/TB co-infected individuals are shown. The graphs are presented as median values (25–75% IQR). Statistically significant differences (*p*-value < 0.05) are indicated.

**Figure 7:**
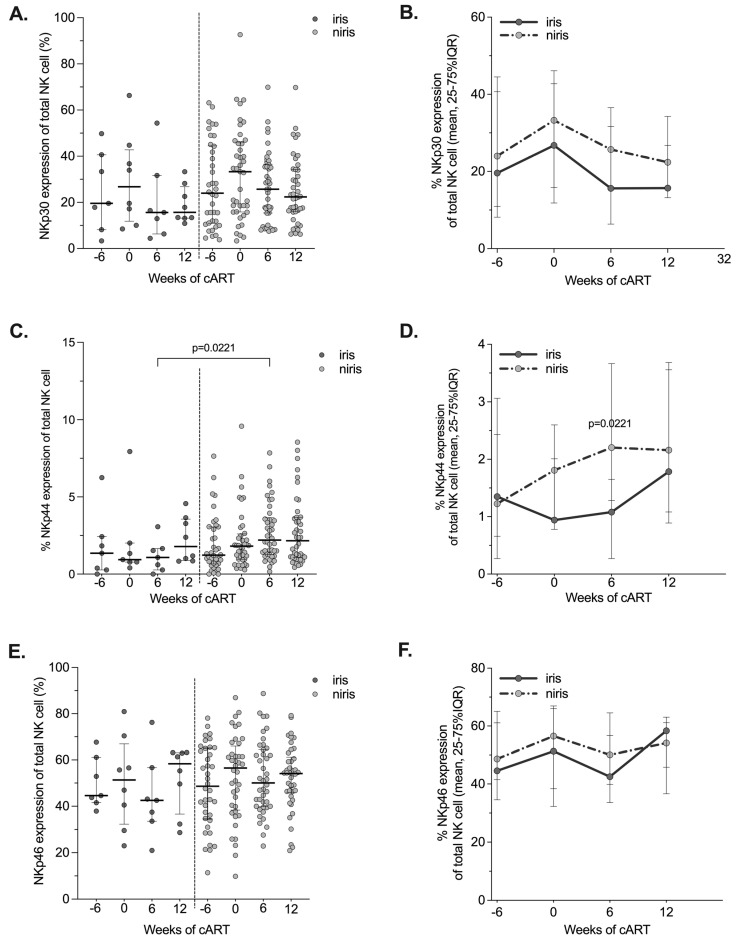
Changes in the expression of NCRs (NKp30, NKp44, NKp46) on NK cells in the late cART introduction group according to the onset of IRIS. Proportion and longitudinal changes of peripheral blood NK cells expressing NCRs: NKp30 (**A**,**B**), NKp44 (**C**,**D**) and NKp46 (**E**,**F**) at the time of enrolment (6 weeks before cART initiation), at week 0, week 6 and week 12 of cART in TB-IRIS and non-IRIS HIV/TB co-infected individuals. Results are shown median values (25–75% IQR). Statistically significant differences (*p*-value < 0.05) are indicated.

Several studies in HIV-infected individuals have reported a large decrease in the expression of NCRs [24,39]. Conflicting results have been published regarding the restoration of NCR expression on NK cells after cART initiation. Therefore, we investigated the reconstitution of the NCR NK cell repertoire after cART initiation in IRIS and non-IRIS individuals in both arms. Although significantly lower expression of NKp30 was observed on NK cells of IRIS subjects in the early arm compared to non-IRIS subjects after six weeks of ARV treatment (Figure 6A and Figure 7A), the kinetics of NKp30^+^NK cells during follow-up were quite similar in both groups, with similar levels at week 32 (Figure 6B). For the other two NCRs (NKp44 and NKp46), no difference in levels or kinetics was observed between IRIS and non-IRIS subjects(Figure 6C–F). In subjects enrolled in the late arm (Figure 7), no difference was observed for the three NCRs, except for the expression of NKp44 after six weeks of cART in non-IRIS subjects, which had higher levels than in IRIS individuals (Figure 7C,D) which may be indicative of a very recent activation of the NK cells in vivo.

## 4. Discussion

This study is the first to investigate NK cell innate immune reconstitution in the context of IRIS in HIV/TB co-infected individuals (CAMELIA clinical trial). Of the 147 HIV/TB co-infected individuals from the CAMELIA trial, we assessed NK cell reconstitution in 37 individuals with confirmed IRIS and compared it with that in 91 individuals without IRIS. We analysed those enrolled in the early and late arms of the trial separately, as the timing of cART initiation was different. In the early arm, we found no differences in the proportion of total NK cells and their subsets between IRIS and non-IRIS individuals either before cART initiation (after two weeks of TB treatment) or during the 32 weeks of follow-up. In contrast, in the late arm, although most of the differences were observed after TB treatment (8 weeks), only the CD56 ^dim^CD16^neg^ NK cell population differed between TB-IRIS and non-IRIS. However, similar proportions of total NK cells and their subsets were observed at the end of follow-up.

Several studies have reported that the total number of NK cells and their subset distribution in peripheral blood are altered in both HIV and active TB infection [22,27,28,40]. However, it has also been reported that the proportion of total NK cells in HIV/TB co-infected individuals with active tuberculosis was similar to that in healthy individuals before and after treatment [40]. An imbalance in NK cell subset distribution has also been observed in TB infection [27,41,42] due to sequestration of the CD56^bright^ NK subset at *M.tb* multiplication sites (i.e., pleural exudate fluid) in TB mono-infected individuals and in a non-human primate model [43,44]. Such changes were at least partially reversed by cART and successful anti-TB treatment [27,28,42,45], and a delayed recovery of peripheral blood NK cell numbers relative to that of CD4 T cells has been reported [46,47]. Our results regarding the evolution of NK cell subsets during cART are in agreement with the other studies [48,49]. Chronic activation of NK cells has been reported in individuals receiving cART for more than ten years with an undetectable plasma HIV viral load [24,25,50], as well as a sustained decrease in some subsets, in particular the CD56^dim^CD16^pos^ subset. The evolution of total NK cells and subsets does not appear to be affected by the onset of IRIS in the early arm and does not differ from that reported by others, either in HIV/TB co-infected individuals or in HIV infection alone.

All NCRs (i.e., NKp30, NKp44, NKp46) tended to be stable over time in the TB-IRIS and non-IRIS individuals regardless of the arm of cART initiation. However, NKp44 expression was higher in non-IRIS than TB-IRIS individuals in the late arm after six weeks of cART. The restoration to normal levels of NCR expression in HIV/TB co-infected individuals has been reported and depended on the AIDS status at the time of cART initiation [26,51]. A lower reconstitution of NCR expression on NK cells could affect the appropriate regulation of innate depend on immune response as DC and antigen presentation in HIV/TB co-infected individuals.

Hyperactivation of the innate immune system and antigen-specific memory CD4 T cells have been reported to be involved in IRIS [7,52,53] and may limit immune recovery. Our results show that CD69 expression decreased continuously and similarly in both IRIS and non-IRIS individuals in the early or late arm, suggesting a good response to the ART regimen. Similarly, decreased levels of the activation marker CD69 on the surface of NK cells have been observed in HIV patients receiving cART [54]. We also observed an increase in the expression of CD122 (the result was not shown) in all patient groups following cART, suggesting at least partial restoration of NK cell homeostasis [55]. We did not observe any changes in the number of NK cells expressing proteins of the KIR family in both IRIS and non-IRIS individuals during the follow-up, as reported by others in HIV individuals [26].

Upregulation of NKG2A expression in HIV-1 infected individuals presenting an advanced clinical status was reported in two studies [56,57], whereas the expression of these markers is not affected in TB individuals [42]. We observed a decrease of NKG2A to similar levels in both IRIS and non-IRIS individuals, associated with an increase of NKG2C levels in both groups. This is consistent with previous results reported for cART-treated HIV individuals [26,58].

NKG2D has been demonstrated to play a role in vitro in NK cell-mediated killing of *M. tb*-infected monocytes via the recognition of ULBP-1 [31]. In TB, it was reported that NKG2D expression was not affected before or after TB treatment [42] and tended to be increased/normal in HIV/TB co-infected and TB mono-infected individuals [59]. We observed stable NKG2D expression that was similar for IRIS and non-IRIS individuals in both arms during the follow-up [37]. However, NKG2D expression on NK cells increases in non-IRIS versus IRIS individuals after six weeks of cART in the early arm as well as in the late arm.

## 5. Conclusions

This is the first study to report NK cell reconstitution in IRIS individuals. Treated HIV/TB co-infected individuals did not reconstitute the NK cell compartment to the same level as treated HIV mono-infected individuals. Despite spatial changes in the repertoire, similar levels of NK cell receptors and/or markers were observed at the end of follow-up, suggesting that NK cell recovery in IRIS was equivalent to that of non-IRIS individuals in the early and late arms. These results highlight the role of coexisting infections and IRIS in the process of innate immunity recovery observed in HIV-infected individuals on cART.

## Data Availability

All datasets generated and analysed in this study are included in the manuscript and Supplementary Files.

## Supplementary Materials

The following supporting information can be downloaded at https://www.mdpi.com/article/10.3390/pathogens12101241/s1, Figure S1: Gating strategy for the analysis of NK cells and their subset and repertoire expression on the cell surface of NK cells. NK cells are defined as the cells that express the markers CD56 and CD16, by analysing them in the CD3 negative gate. The subsets of NK cells are identified by the expression pattern of CD56 on their cell surface. The proportion of CD56posCD16pos NK cells in the total NK cell gate is analysed for the expression of the NK cell repertoire. Figure S2: Changes in the expression of activation marker CD69 and Killer immunoglobulin-like receptors (KIR CD158a, KIR CD158i) on NK cells in the Early cART initiation group according to the onset of IRIS. The proportion and longitudinal changes of peripheral blood NK cells expressing CD69 (A and B), KIRs CD158a (C and D) and NKp46 (E and F) at the time of enrolment (week 0), week 6, 12 and week 32 of cART in TB-IRIS and non-IRIS HIV/TB coinfected individuals are shown. The graphs are presented as median values (25–75% IQR). Statistically significant differences (*p*-value < 0.05) are indicated. Figure S3: Changes in the expression of activation marker CD69 and Killer immunoglobulin -like receptors (KIR CD158a, KIR CD158i) on NK cells in the Late cART initiation group according to the onset of IRIS. The proportion and longitudinal change of peripheral blood NK cells expressing CD69 (A and B.), KIRs CD158a (C and D.) and KIRs CD158i (E. and F.) at the time of enrolment (6 weeks before cART initiation), at week 0, week 6 and week 12 of cART in TB-IRIS and non-IRIS HIV/TB co-infected individuals. Results are presented as median values (25%–75% IQR). Statistically significant differences (*p*-value < 0.05) are shown. Figure S4: Changes in the total number of NK cell counts and their subsets in the early cART introduction group (Early arm) by IRIS occurrence. Longitudinal evolution of total circulating NK cells (A) and their subsets: CD56brightCD16neg (B), CD56dimCD16neg (C), CD56dimCD16pos (D), CD56negCD16pos (E) NK cells at baseline (week 0) and after 6, 12, 32 weeks of cART in TB-IRIS and non-IRIS HIV/TB coinfected individuals. Results are presented as median values (25–75% IQR). The *p*-value <0.05 is statistically different significant. Figure S5: Changes in the total number of NK cells and their subsets in the late cART initiation group (Late arm group) according to the onset of IRIS. Longitudinal evolution in peripheral blood NK cells (A) and their subsets: CD56brightCD16neg (B), CD56dimCD16neg (C), CD56dimCD16pos (D), CD56negCD16pos (E) NK cells at baseline (week 6) and at 0, 6, 12 weeks of cART in TB-IRIS and non-IRIS HIV/TB coinfected individuals. Results are presented as median values (25–75% IQR). The *p*-value < 0.05 is statistically different significant. Table S1: List of monoclonal antibodies for NK repertoire immune-phenotyping assay by flow cytometry.

## Appendix A. List of Study Team Members and Investigators in Cambodia

**CAMELIA study team members**: Thim SOK, François-Xavier BLANC, Anne E. GOLDFELD, Didier LAUREILLARD, Claire REKACEWICZ, and Sirenda VONG.
**CAMELIA coordination centre**: Phearavin PHENG, Manil SAMAN, Chanthy LENG, Sao Sarady AY, Phalla CHEA, Nimul Roat MEN, Sopheap KUN, Sokeo CHEA, Pichda TOUENG, Yong YOEUN, Keo Kunthea DY, Pheakun KRY, and Keolinelyanneth MEARDEY.
**Khmer Soviet Friendship Hospital**: Narom PRAK, Chhun Im SIN, Chan Chhaya NGET, Sor KAING, Sovannara SONG, Bora AM, Sok CHEUNG, Tuon Seang Y, Seng Ly SAY, Sarann SUM, Seam CHHUN, Vanny SAY, Sokhoeun KAM, Chanthan UNG, TE Nai Sim, Sokim CHHOEUN, Chuop Chum KOH, Ratanak TONG, Cheung SOK, Saorin TITH, and Sithan CHAN.
**Svay Rieng Hospital**: Kim khemarin LAK, Sath SUN, Sean YIM, Nimul CHAN EAR, Dara CHAN, Sokbophang VOEUNG, Phally BE, Chan Nimul EAR, Vanda PRAK, Thoeurn MEN, Thoeun KOY, Somaly THACH, Sinan CHHAY, and Sina MEAS.
**Donkeo Hospital**: Chindamony KIM, Thong PHE, Saroeun UORNG, Sokhon KUOCH, Phea LAO, Chanthan YIM, Samros KHIEV, Sareth HEM, KEO Kimthan, UM Samoeun, TEANG Sophary, MEY imtheavy, Chanthon YIM, Bunthoeun HEANG, Hort SIENG, Huch MEAS, Sina SOK, Vibol NEAK, Nyn KEN, Kunthea TES, Soeun SAN, and Soeun SEAN.
