# Supplementary material for: Natural Killer Repertoire Restoration in TB/HIV Co-Infected Individuals Experienced an Immune Reconstitution Syndrome (CAMELIA Trial, ANRS 12153)
Source: Pathogens. 2023 Oct 13;12(10):1241. doi: 10.3390/pathogens12101241 (PMC10610037; doi:10.3390/pathogens12101241)
Supplement: Supplementary file 1 [file pathogens-12-01241-s001.zip › Tab-s1.pdf]

Supplementary table S1.

A. Fluorchrome-conjugated monoclonal antibodies use for NK cell repertoire and functional analysis

N^0	Marker	CD nomenclature	Conjugated fluorochrome	Clone	Cat #	Manufacturer
1	CD3	CD3	APC	UCHT1	IM2467	Beckman Coulter
2	NCAM	CD56	PC5	N901 (NKH-1)	A79388	Beckman Coulter
3	FcγR III	CD16	PC5	3G8	A07767	Beckman Coulter
4	FcγR III	CD16	FITC	3G8	IM0814U	Beckman Coulter
6	CD69	CD69	PE	TP.1.55.3	IM1943U	Beckman Coulter
7	NKG2A	CD159a	PE	Z199	IM3291U	Beckman Coulter
8	NKG2D	CD314	PE	ON72	A08934	Beckman Coulter
9	ILT2	CD85j	PE	HP-F1	A07408	Beckman Coulter
10	NKp44	CD336	PE	Z231	IM3710	Beckman Coulter
11	NKp46	CD335	PE	BAB281	IM3711	Beckman Coulter
12	NKp30	CD337	PE	Z25	IM3709	Beckman Coulter
13	KIR2DL1, KIR2DS1	CD158a,h	PE	EB6B	A09778	Beckman Coulter
14	KIR2DL2, KIRD2DL3, KIR2DS2	CD158b1, b2, j	PE	GL183	IM2778U	Beckman Coulter
15	KIR3DL1, KIR3DS1	CD158e1/e2	PE	Z27.3.7	IM3292	Beckman Coulter
16	KIR2DS4	CD158i	PE	FES172	IM3337	Beckman Coulter
17	IL-2Rbeta	CD122	PE	CF1	IM1978	Beckman Coulter
18	2B4	CD244	PE	C1.7	IM1608	Beckman Coulter
19	KLRD1	CD94	PE	HP-3B1	IM2276	Beckman Coulter
20	CD160	CD160	PE	BY55	IM3657	Beckman Coulter
21	NKR-P1A	CD161	PE	191B8	IM3450	Beckman Coulter
22	LAMP-1	CD107a	FITC	H4A3	555800	Beckton Dickinson
23	IFN-gamma	IFN gamma	PE	25723.11	340452	Beckton Dickinson
24	KLRF1	NKp80	PE	239127	FAB1900A	R & D System
25	NKG2C	CD159c	PE	134591	FAB138P	R & D System
26	DNAM-1	CD226	PE	102511	FAB666P	R & D System

B. Combination of fluorochrome-conjugated monoclonal antibodies for immune-phenotyping

1). NK cell repertoire phenotyping

Nº	APC	PC5	FITC	PE
1	CD3	CD56	CD16	CD69
2	CD3	CD56	CD16	NKp44
3	CD3	CD56	CD16	NKp46
4	CD3	CD56	CD16	NKp30
5	CD3	CD56	CD16	NKp80
6	CD3	CD56	CD16	CD158a/h
7	CD3	CD56	CD16	CD158b1/b2/j
8	CD3	CD56	CD16	CD158e
9	CD3	CD56	CD16	CD158i
10	CD3	CD56	CD16	NKG2A
11	CD3	CD56	CD16	NKG2D
12	CD3	CD56	CD16	CD94
13	CD3	CD56	CD16	NKG2C
14	CD3	CD56	CD16	CD85j
15	CD3	CD56	CD16	CD122
16	CD3	CD56	CD16	CD244
17	CD3	CD56	CD16	CD160
18	CD3	CD56	CD16	CD161
19	CD3	CD56	CD16	DNAM-1

2). NK cell cytotoxic and cytokine intracellular staining

Nº	APC	PC5	FITC	PE
1	CD3	CD56+CD16	CD107a	IFN-gamma
