# Supplementary figures and images for: Natural Killer Repertoire Restoration in TB/HIV Co-Infected Individuals Experienced an Immune Reconstitution Syndrome (CAMELIA Trial, ANRS 12153)
Source: Pathogens. 2023 Oct 13;12(10):1241. doi: 10.3390/pathogens12101241 (PMC10610037; doi:10.3390/pathogens12101241)

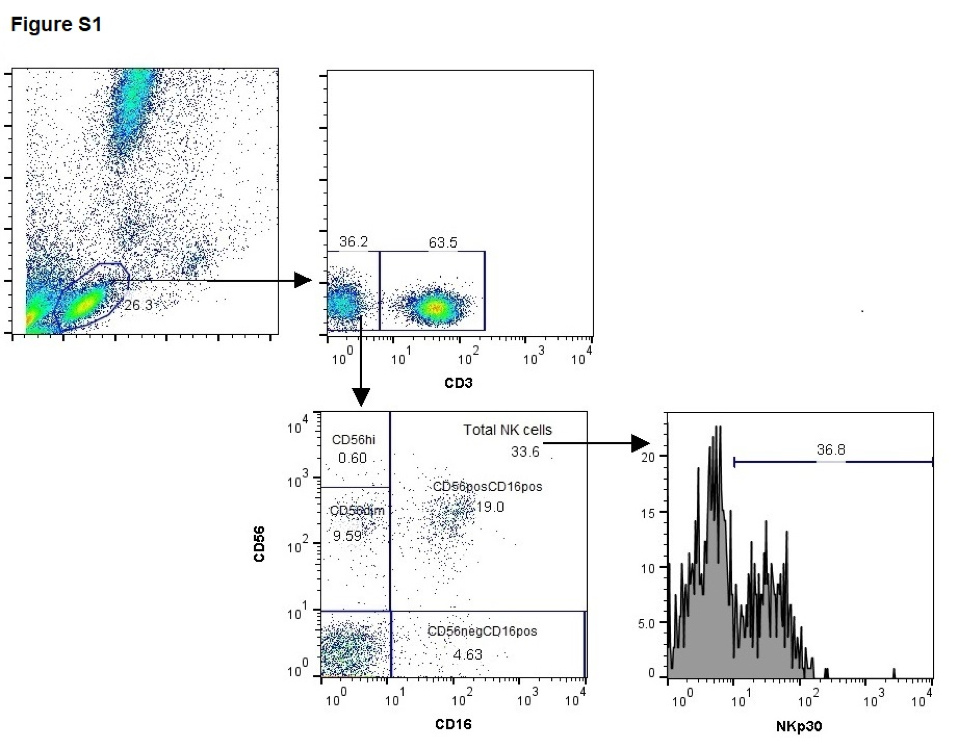

Supplement: Supplementary file 1 [file pathogens-12-01241-s001.zip › Figure s1.png]

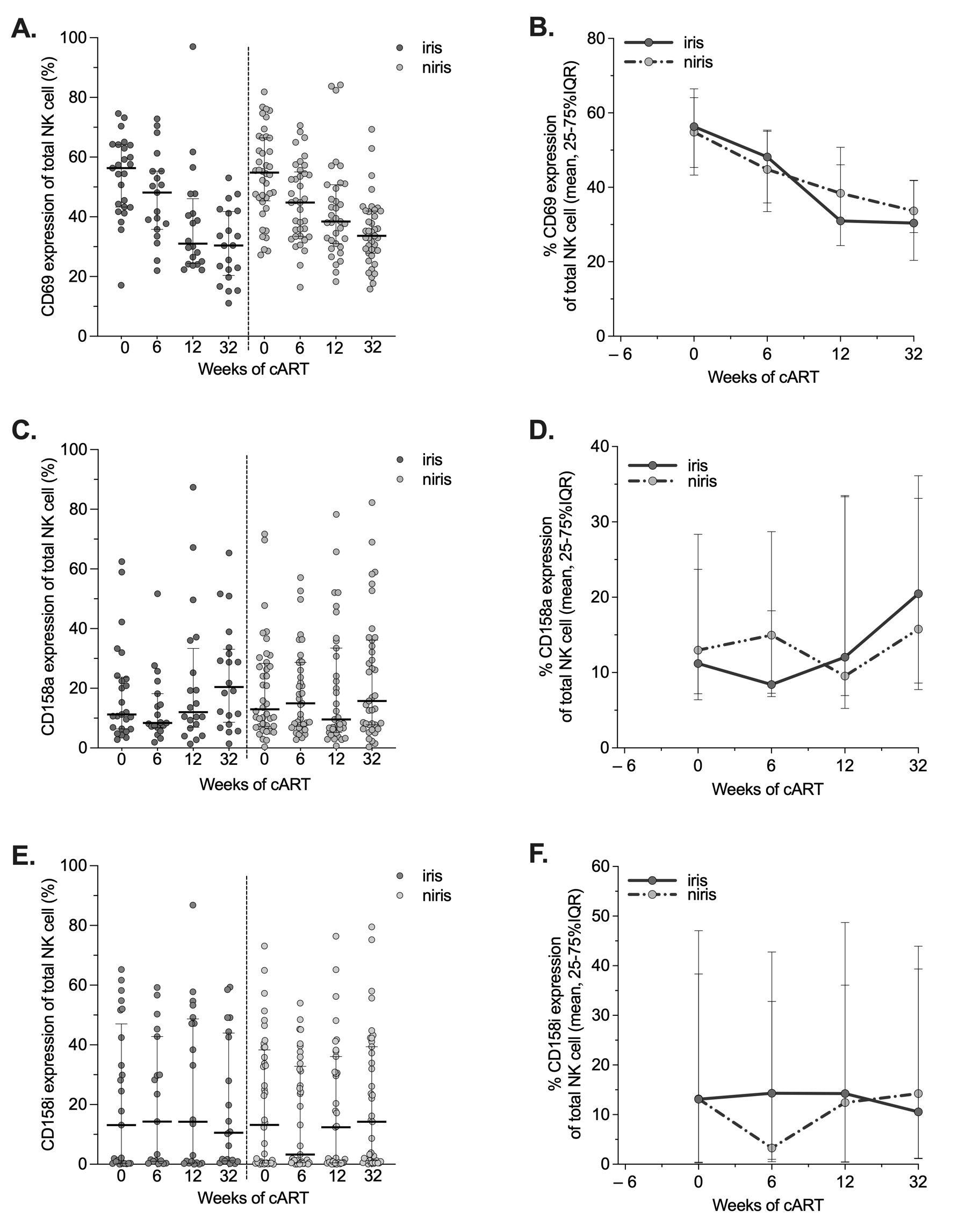

Supplement: Supplementary file 1 [file pathogens-12-01241-s001.zip › Figure s2.tiff]

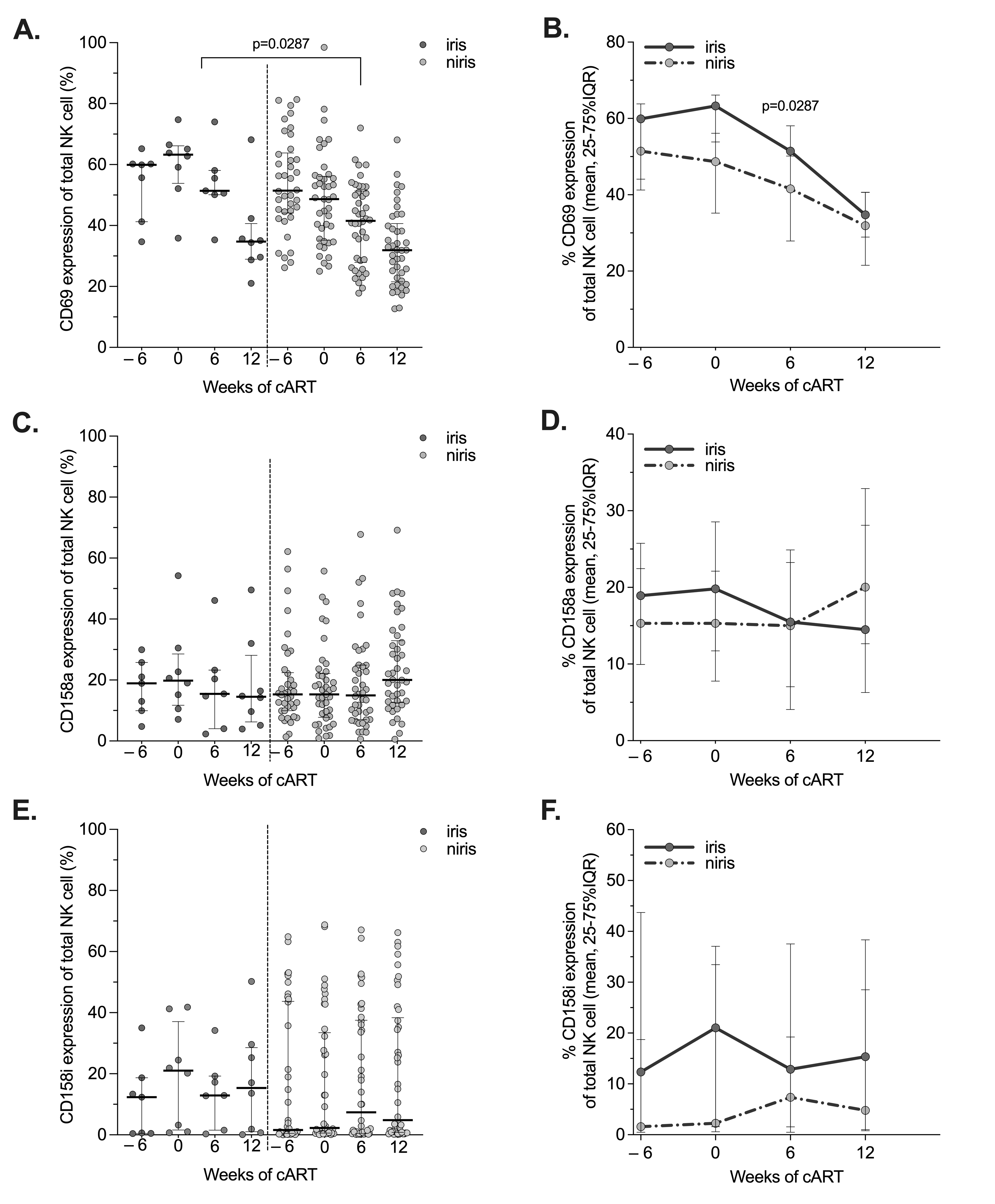

Supplement: Supplementary file 1 [file pathogens-12-01241-s001.zip › Figure s3.tiff]

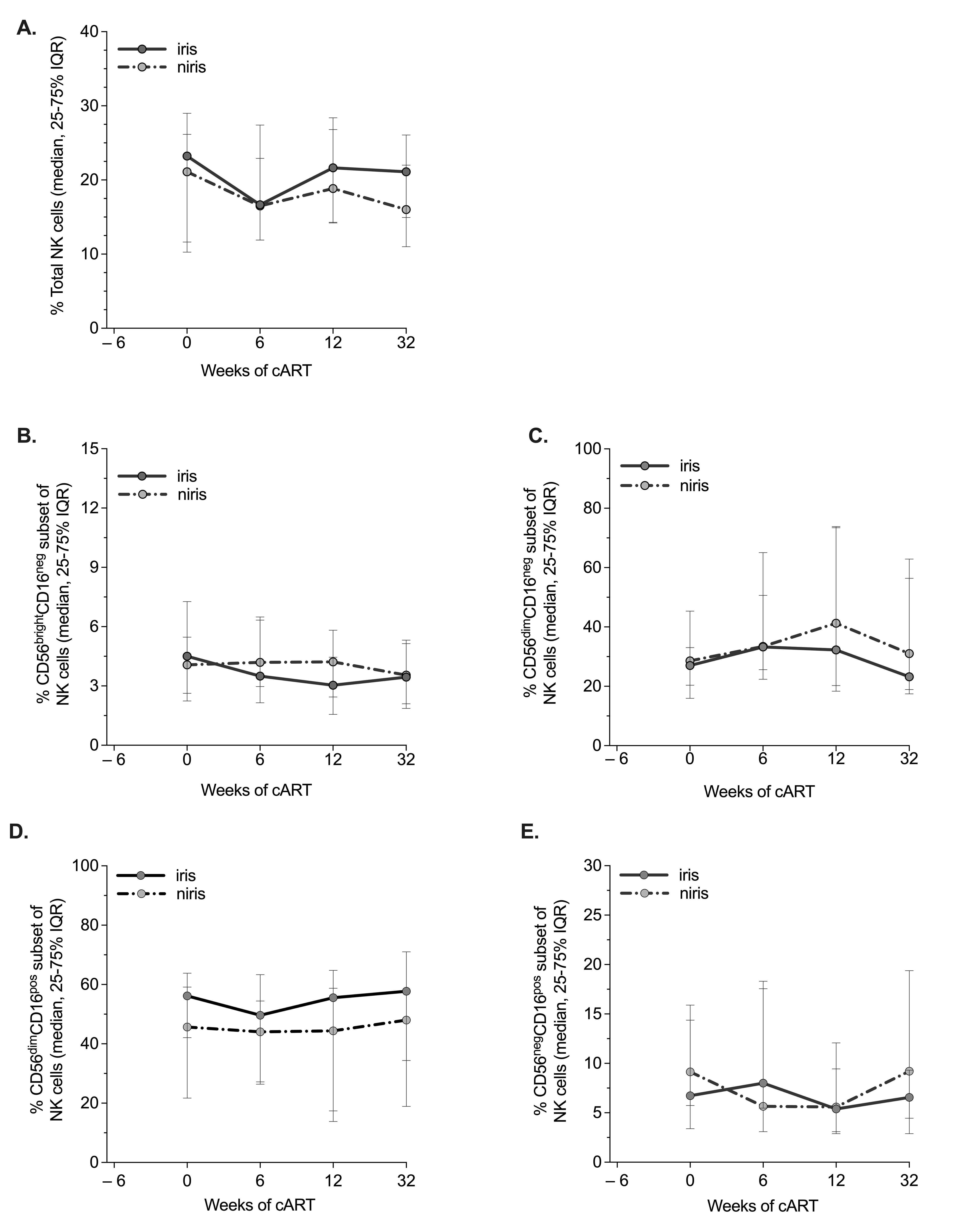

Supplement: Supplementary file 1 [file pathogens-12-01241-s001.zip › Figure s4.tiff]

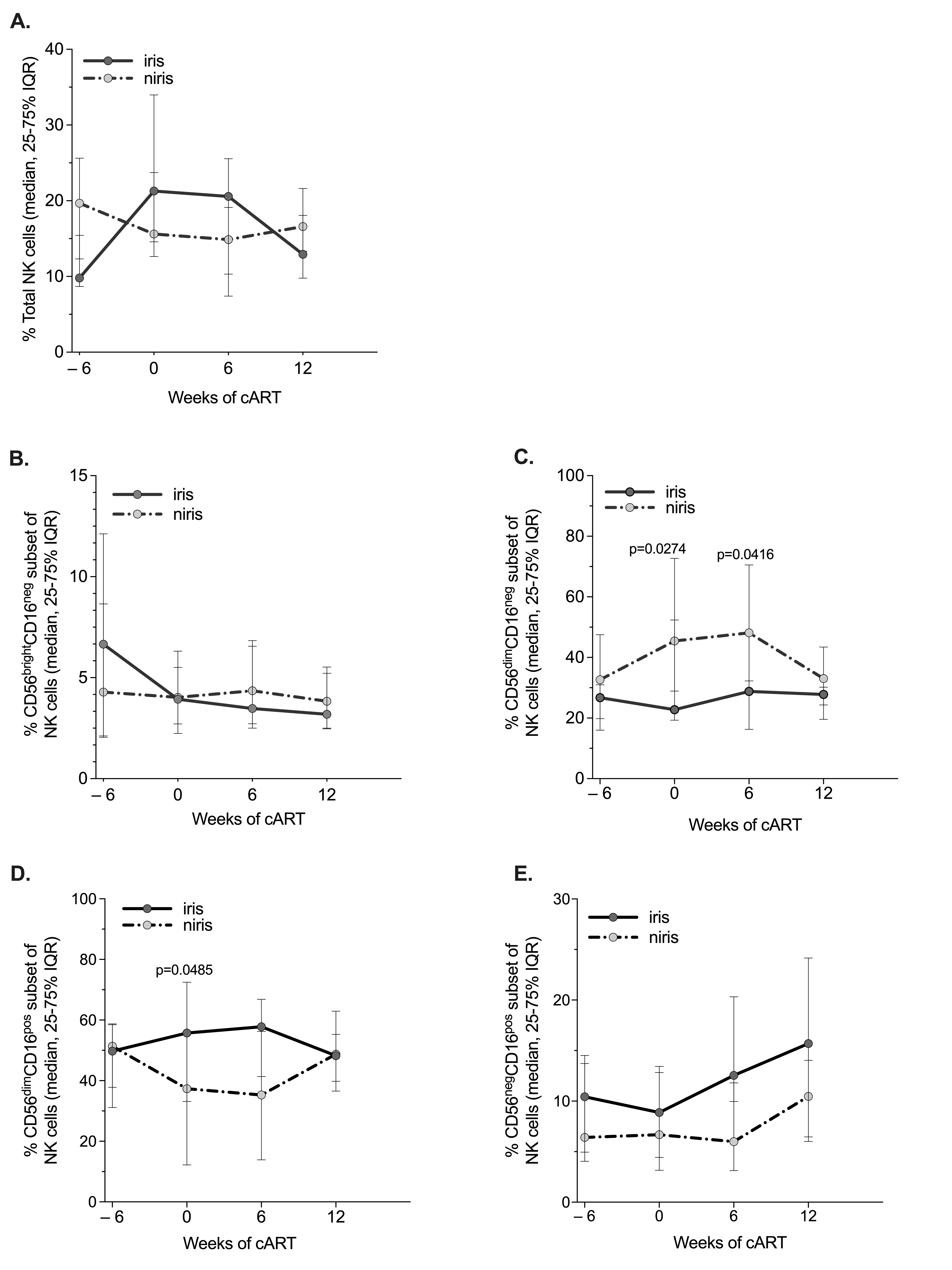

Supplement: Supplementary file 1 [file pathogens-12-01241-s001.zip › Figure s5.tiff]
